# Role of Aldynoglia Cells in Neuroinflammatory and Neuroimmune Responses after Spinal Cord Injury

**DOI:** 10.3390/cells10102783

**Published:** 2021-10-17

**Authors:** Vinnitsa Buzoianu-Anguiano, Mabel Torres-Llacsa, Ernesto Doncel-Pérez

**Affiliations:** 1 Grupo de Química Neuro-Regenerativa, Hospital Nacional de Parapléjicos, SESCAM, 45071 Toledo, Spain; vbuzoianu@externas.sescam.jccm.es; 2 Servicio de Radiología, Hospital Nacional de Parapléjicos, SESCAM, 45071 Toledo, Spain; mabelt@sescam.jccm.es

**Keywords:** aldynoglia, axonal growth, bdnf, ensheathing cells, epscs, gfap, macrophage, microglia, p75 ngfr, sci, vimentin

## Abstract

Aldynoglia are growth-promoting cells with a morphology similar to radial glia and share properties and markers with astrocytes and Schwann cells. They are distributed in several locations throughout the adult central nervous system, where the cells of the aldynoglia interact and respond to the signals of the immune cells. After spinal cord injury (SCI), the functions of resident aldynoglia, identified as ependymocytes, tanycytes, and ependymal stem cells (EpSCs) of the spinal cord are crucial for the regeneration of spinal neural tissue. These glial cells facilitate axonal regrowth and remyelination of injured axons. Here, we review the influence of M1 or M2 macrophage/microglia subpopulations on the fate of EpSCs during neuroinflammation and immune responses in the acute, subacute, and chronic phases after SCI.

## 1. Introduction

In the central nervous system (CNS), the activity of glial cells plays an essential role. They act like cellular support and constituting a source of growth factors in the CNS. After CNS injury, glial cells promote cell survival and neurogenesis in adults. Astroglial cells participate in the tripartite synapse as the third cellular component of pre- and postsynaptic neurons and act as a key regulator at the synapse [1]. Oligodendroglial cells ensure synaptic transmission by enveloping axons and producing myelin [2]. The function of aldynoglia, a glial cell subtype, promotes axonal growth, ensheathing, and myelination of neurons [3] and has been used in spinal cord injury models [3,4,5].

The close relationship between glial cells and neurons is of great importance for the functioning of CNS, both for myelination and the transmission of neurotransmitters, even if they come from a different progeny of precursor cells [6]. Neurons and glial cells come from neuroectoderm, the precursors of glial cells will be found in both the ventricular and subventricular areas from where they migrate to more specific regions using neuron-like signals [7].

The highest percentage of glial cells in the brain are astrocytes and oligodendrocytes, but there are other types of glial cells such as radial glia, Müller glia cells, Bergman glial cells, pituicytes, tanycytes, and olfactory ensheathing cells. In addition, in the spinal cord are the ependymal cells, tanycytes, and cells of the central canal; together they are called aldynoglia cells, which remain in adulthood [6,7].

## 2. Characteristics of Aldynoglia Cells in Brain

**Figure 1:**
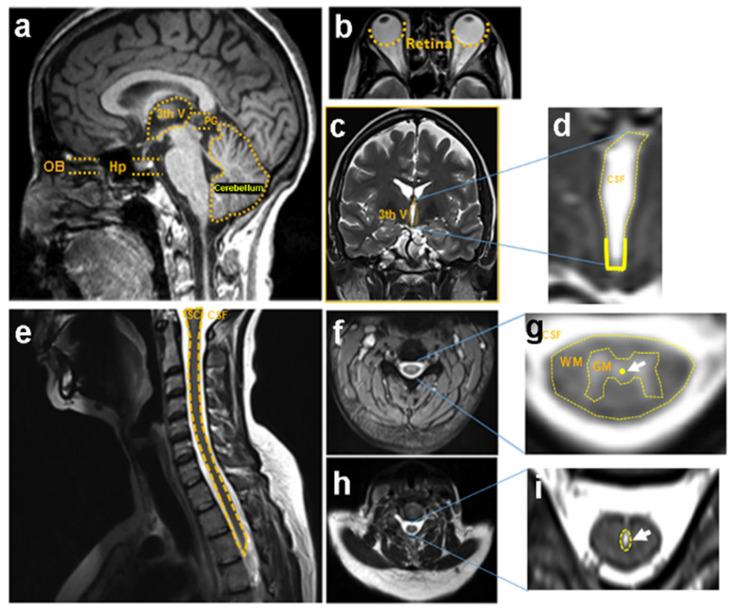
Locations of the aldynoglia cell subpopulation in the human CNS. The magnetic resonance imaging show neural niches for aldynoglia in the brain (**a**–**d**) and spinal cord (**e**–**i**): olfactory bulbs, hypophysis, third ventricle, pineal gland, cerebellum in (**a**), and both retinas (**b**). A coronal section of MRI showing the lateral ventricles (**c**) with enlarged third ventricle filled with cerebrospinal fluid (**d**), high presence of aldynoglia cells in the floor, and the lateral walls are marked. Spinal cord as a broad zone for aldynoglia, sagittal MRI section of the cervical zone of SC floating in CSF is shown (**e**). The axial section at C7 level (**f**) is enlarged showing the medullar channel with CSF surrounding white matter and grey matter, which contains aldynoglia cells (**g**). Arrows pointed to the location of resident aldynoglia in the ependyma of a normal individual (**g**) or activated aldynoglia in widened ependyma with hydromyelia in the spinal cord of patient (**i**). 3th V, third ventricle; C7, seventh vertebral cervical level; CSF, cerebrospinal fluid; GM, grey matter; Hp, hypophysis; MRI, magneting resonance imaging; OB, olfactory bulbs; PG, pineal gland; SC, spinal cord; WM, white matter.

Aldynoglia subpopulation cells were described in Nieto-Sampedro lab [8]; they are CNS cells and can be found in the human brain in: olfactory bulb, hypothalamus, third ventricle, pineal gland, retina, and cerebellum (Figure 1a–d). They have similar properties to the Schwann cells of the peripheral nervous system (PNS) and astrocytes and have the capacity for proliferation and survival; they also promote neuritic growth and myelination [8]. These aldynoglia cells share their ontogenic origin with astroglia and have immunoreactivity for glial fibrillary acid protein (GFAP), S100β, and p75 nerve growth factor receptor (p75 NGFR) like Schwann cells and astrocytes (Table 1) [5,7].

Other physiological properties of aldynoglia are its ability to wrap non-myelinated axons and the favoring of axonal growth, which may explain the regeneration in CNS of olfactory and hypothalamic axons [5,7,8]. Its characteristics, from the point of view of its cell biology, show typical traits of developing cells and have an immature cytoskeleton, present a positive marking for vimentin and the estrogen receptor α-type, ERα (Table 1) [7,9].

## 3. Morphological and Functional Characteristics of Tanycytes and Pituicytes

The tanycytes are found in the ventrolateral wall and floor of the third ventricle in the adult brain (Table 1; Figure 1); their radial appearance helps axons during development as a guide for release of the hormone gonadotropin (GnRH) from the glial processes into neurons; they act as transporters of hormones. The tanycytes found in median eminence can carry β-endorphins from the neuron to the cerebrospinal fluid [9,10].

In the case of pituicytes, these cells are non-neuronal elements of the neural or posterior lobe, neurohypophysis, in the hypothalamus (Table 1, Figure 1). They surround axonal endings and regulate hormone secretion by releasing their processes from these endings [18], and their cytoplasm presents specific intermediate filaments made up of GFAP [19].

## 4. Olfactory Ensheathing Cells

The olfactory ensheathing cells are glial cells of the primary olfactory system and are located in the olfactory nerve, cranial nerve I (Table 1; Figure 1). It extends between the olfactory epithelium of the root of the olfactory cavity and the olfactory bulb in the anterior cranial cavity, from the lamina of the olfactory mucosa to the inner and outer layers of the olfactory nerve fibers [11]. These cells support regeneration by promoting cell–cell interaction and improving the microenvironment for axonal growth, as they act as substrates for axonal guide and promote cell restoration. In addition, the ensheathing cells can modulate the immune system, provide neuroprotection, angiogenesis; and they secrete growth factors such as brain-derived growth factor (BDNF), nerve growth factor (NGF), and neurotrophin 3 (NT-3). Moreover, these cells wrap axons for remyelination and secrete molecules to the extracellular matrix that facilitate the growth of new axons [4].

## 5. Müller Cells

Müller’s cells are the primary glial radial cells specializing in the retina, encompassing the neural retina’s thickness, from the surface of the lens to the subretinal space (Table 1; Figure 1). These subpopulations of aldynoglia cells also express p75 NGFR, GFAP, and vimentin [12,13]. They differ from the radial glial cells of the cortex as they do not act as progenitor cells, nor do they serve as a matrix for neuron migration during retinal development, but they maintain characteristics such as progenitors, expressing progenitor-like genes, and can proliferate and generate neurons under certain conditions [20]. The Müller cells act as a support for the metabolism of the retina neurons; they provide trophic substances to the neurons and are capable of removing metabolic waste [21].

## 6. Bergmann Glial Cells

The cerebellum is a brain region that plays an essential role in motor control, mainly in movement coordination and accuracy (Table 1; Figure 1). The cerebellar cortex of mammals is composed of three layers: the molecular outer layer containing the axons of granular neurons, the dendrites of Purkinje cells, the fibers of Bergmann’s glial cells, basket cells, and stellated cells. The second is the Purkinje cell medial layer containing Purkinje cells, Bergmann’s gloss cells; and the third is the granular internal cell layer containing Golgi’s granular cells [14,22].

Each Bergmann glial cell extends two to six fibers to the sub-pial basal membrane; its fibers aid the migration of neurons and elongation of dendrites and axons. In the mature cerebellum, it actively participates in processing cerebellum information and can express cellular markers of the stem, such as SOX 1, SOX 2, and SOX 9, giving them the characteristic of being a trunk cell in the adult cerebellum, also can be expressed GFAP and vimentin [14]. Their role is in corticogenesis, including in foliation, cortical layer assembly, act as a guide and support for neuron migration, neurotic growth, and regulating synaptogenesis. They constitute a physical scaffold promoting trophic support and regulating signals that modulate the different ontogenic processes in all these processes [23].

## 7. Pineal Gland Cells

The pineal gland is the organ that is responsible for carrying out and regulating the Circadian cycle by transmitting light from the environment as an internal signal, melatonin (Figure 1). The pineal gland develops from an area of the neuroepithelium close to the roof of the third ventricle [24,25]. The pineal gland is performed by a collection of cells, mainly pinealocytes (associated with melatonin); you can also find astrocytes, microglia, and endothelial cells. Pinealocytes have been divided into two types: Type I comprises 90% of the parenchyma cells; their morphology is long with a euchromatic nucleus and a prominent nucleolus. Type II represents around 15% of the cells; these are smaller with an oval nucleus and denser [24,25]. The characteristic of pinealocytes are the markers that they share with the cells of the aldynoglia, and they have been shown to be positive for GFAP, vimentin, S100β (Table 1) [15,16,17].

## 8. Aldynoglia Cells in Spinal Cord

In the spinal cord, we can find aldynoglia cells as part of the ependymal layer of the spinal cord (SEL) occupying the central area of grey matter in the spinal parenchyma throughout the entire spinal cord (Table 2; Figure 1). Within SEL, we find tanycytes and ependymocytes as glial cells and the neurons of the central canal [26]. These glial cells, like those of the brain, share glial markers such as vimentin and S100β. In addition, they express nestin, an intermediate filament protein expressed in dividing cells during the early stages of development in the CNS and peripheral nervous system. Nestin is reinduced in the adult during pathological situations, such as the formation of the glial scar after CNS injury [27,28]. The GFAP marker is not co-expressed with nestin or vimentin in these aldynoglia cells; however, it has been reported that ependymocytes can differentiate between GFAP+, S100β+ cells after spinal cord injury, functioning like an astroglial precursor cell, Table 2 [27,28,29,30].

The cells of the ependymal layer, from the embryological point of view, originate from cells of the neuroepithelium of the neural plate and have characteristics of progenitor cells [26,31]. These cells of the ependymal layer are cuboidal homogeneous monolayer cells that consist of three types of cells: ependymocytes, tanycytes, and neurons in contact with cerebrospinal fluid [26,31]. Ependymocytes are the primary cell type of the SEL layer, and they have a cuboidal epithelial morphology and 1 to 4 luminal cilia. These cells act as structural support for the central canal of the spinal cord [26,31].

On the other hand, tanycytes are radial glial cells with simple luminal cilia; these cells carry out the bioactive transport of substances between the cerebrospinal fluid (CSF) and the blood vesicles. It has been demonstrated that these cells secrete a vasoactive peptide that favors the control of the vascular tone of the spinal cord [26,31]. Activated aldynoglia, like ependymocytes and tanycytes (Table 2), has been detected as GFAP positive cells around the central canal. These aldynoglia could be associated with a widening of the central canal observed in some pathological conditions like idiopathic hydromyelia or post-traumatic syringomyelia [32].

Neurons in contact with cerebrospinal fluid are a population of cells with a morphology similar to the retina’s unipolar neurons. They are distributed around the central canal, adjacent to the cuboidal layer of the ependymal cells also found in the spinal cord’s terminal filum. These neurons’ function is possibly sensory in type, as they can process changes in the composition of cerebrospinal fluid, its pressure, and its flow. Also, they present receptors for different molecules such as acetylcholinesterase, GABA, vasoactive intestinal peptide, somatostatin, and ATP receptors such as P2X2. When a change in pH occurs in CSF, these cells can contribute to CSF homeostasis [26]; these sensory functions are maintained thanks to the support of neighboring glial cells.

## 9. Secondary Cell Death in Spinal Cord after Injury

**Figure 2:**
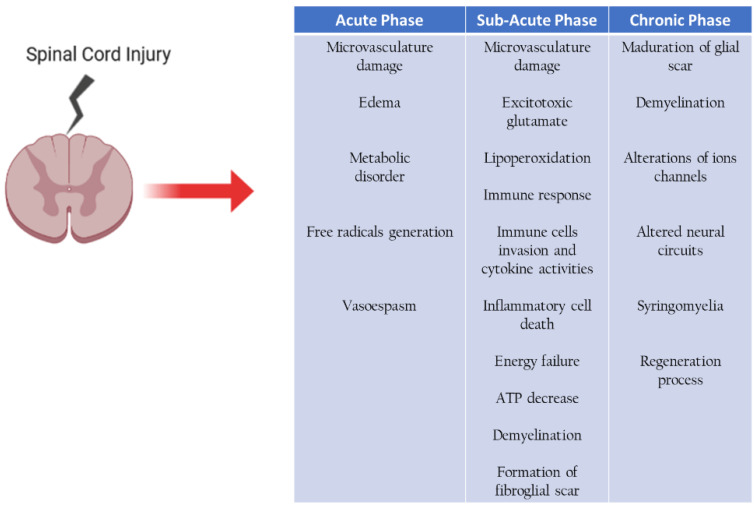
The processes that provokes secondary mechanisms of cell death in the spinal cord after injury.

Traumatic spinal cord injury is a complex pathology resulting from mechanical damage to the spinal cord that can be partial or total, causing loss of sensory and motor functions, becomes irreversible. Two critical events can occur, the primary injury caused by mechanical damage with bleeding, damage to axons and glial cells resulting in cell death at the site of necrosis injury; and secondary injury that triggers different mechanisms of cell death [33,34], (Figure 2).

The second injury is divided into three phases:

- Acute phase, spanning 0 to 2 days after the injury,
- Subacute phase, 3 days to weeks after the injury,
- Chronic phase, 7 weeks to years after the injury,

In the acute and subacute phases, there is damage to the tissue microvasculature and edema, causing a metabolic disorder, thus generating free radicals. There is excitotoxicity by glutamate lipoperoxidation and immune response and initiates the formation of the fibroglial scar. The chronic phase continues the maturation of the fibroglial scar formation, demyelination, and begin proper regeneration processes [34,35], (Figure 2). The immune response is one of the essential mechanisms; its main objective is to stop the degeneration of adjacent healthy tissue, performing the cleansing of cell remains of cell death by necrosis, which leads to a toxic environment for axonal growth.

In the acute phase of SCI in humans, there is a systemic suppression of the immune system by the CNS, known as SCI-induced immunodeficiency syndrome (SCI-IDS) [36]. The SCI-IDS makes patients with SCI lesions highly susceptible to infection and the severity depends on the level of spinal cord injury [37]. SCI-IDS is induced by the CNS through the hypothalamic-pituitary-adrenal axis that regulates the release of glucocorticoids from the adrenal glands, among other mechanisms [38]. It reflects how the CNS modulates the immune response in a detrimental way from this initial phase.

## 10. Innate Immune Response in Spinal Cord Injury

**Figure 3:**
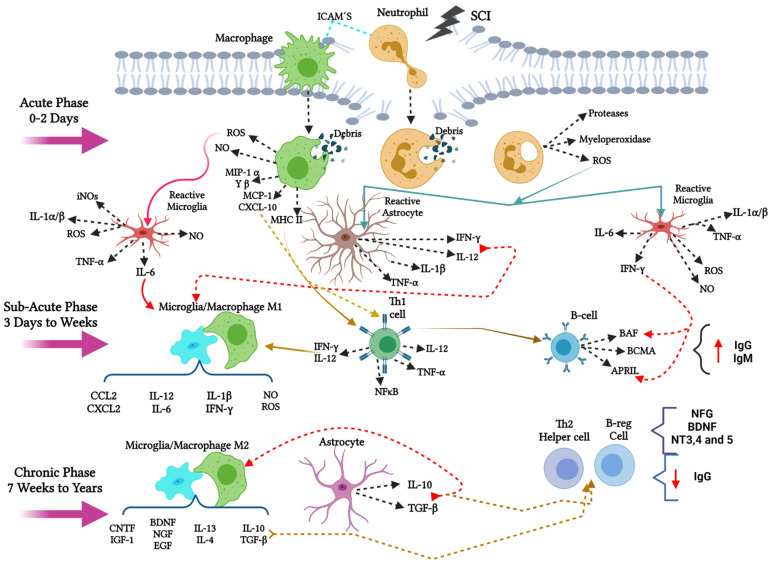
Cellular and molecular components in the three phases of inflammation after a spinal cord injury. After SCI, the first cells to arrive are the neutrophils and later the macrophages; these clean the area of injury and secrete different molecules (Proteases, myeloperoxidase), chemokines (CXCL-10), and reactive oxygen species (ROS). ROS activates microglia and astrocytes, secrete interleukins and more reactive oxygen species, promoting a pro-inflammatory environment. In the sub-acute phase, macrophages and microglia are found in the M1 phenotype and secrete interleukins (IL-6, IL-12, IL-1β) and chemokines (CCL2, CXCL2) that maintain the pro-inflammatory microenvironment. Also to the area of injury are the Th1 cells, which also help this inflammatory environment by secreting interleukins (IL-12, TNF-α, IFN-γ, and secrete antigens recognized by B cells, increasing the system production of autoantibodies. In the chronic phase, astrocytes secrete anti-inflammatory interleukins (IL-10, TGF-β), which produce a balance by changing microglia and macrophages to an anti-inflammatory effect M2, promoting neuroprotection to the surviving cells by the secretion of growth factors (BDNF, NGF, EGF, CNTF, IGF-1) and anti-inflammatory interleukins (IL-13, IL-4, IL-10, TGF-β). This anti-inflammatory microenvironment causes naïve T lymphocytes to become activated in the Th2 helper fate increasing this neuroprotective microenvironment, and therefore B cells decrease autoantibody production.

After the initial damage of a spinal cord injury (SCI), there is an infiltration of macrophages and neutrophils into the injury site for phagocytosis of cellular debris and myelin. In the acute phase, neutrophils secrete molecules such as proteases, myeloperoxidase, or reactive oxygen species, ROS (Figure 3). ROS activate microglia and astrocytes at the site, changing their phenotype to reactive cells. In addition, after phagocytosis, macrophages secrete molecules such as ROS, nitric oxide (NO), macrophage inflammatory protein MIPα and β; monocyte chemoattractant protein 1 MCP-1; CXCL-10 and act as antigen-presenting cells activating the type II histocompatibility complex MCH-II [39].

The secreted ROS activate the resident microglia in the area in its reactive form. Activated microglia secretes interleukins such as interleukin 1 alpha/beta (IL-1α and β); IL-6; tumor necrosis factor-alpha (TNF-α); interferon-gamma (IFN-γ) and reactive oxygen species ROS and inducible nitric oxide synthase iNOS promoting a pro-inflammatory microenvironment. In the same way, astrocytes also secrete interleukins such as IL-12, IFN-γ, IL-1β, TNF-α, creating a pro-inflammatory environment in the area of injury [40,41,42].

## 11. The Adaptive Immune Response after SCI

In the sub-acute phase, interleukins such as IL-6, IFN-γ, and IL-12, promote the rearrangement of the cytoskeleton by polymerization in macrophages/microglia towards a pro-inflammatory phenotype M1, which secrete chemokines CCL2, CXCL2, and interleukins. IL-12, IL-6; IL-1β, IFN-γ; and molecules such as NO and ROS, maintaining an inflammatory environment. In addition, there is a migration of T and B cells towards the site of injury. Th1 effector lymphocytes are activated in the context of MHC-II and CXCL-10 chemokine; when they reach the lesion area, they maintain an inflammatory state and secrete antigens, activating signaling pathways such as nuclear factor-kappa b (NFκB), and promoting cell death. These antigens expressed by T lymphocytes are recognized by B cells, activating molecules such as B cell activating factor (BAF), B cell maturation antigen (BCMA), and proliferation induction ligand (APRIL), encouraging an increase in IgG and IgM autoantibodies in the environment [39,41], (Figure 3).

There is a balance in the system in the chronic phase, promoting change from pro-inflammatory to the anti-inflammatory environment. In this phase, interleukins such as IL-10 and the transforming factor beta TGF-β that, on the one hand, favor polymerization in the M2 macrophage/microglia phenotype and promote the activation of nave lymphocytes in Th2 helpers. The M2 Macrophages/microglia secrete anti-inflammatory cytokines such as IL-10, TGF-β, IL-13; IL-4 and growth factors such as brain-derived growth factor (BDNF), nerve-derived growth factor (NGF), epidermal growth factor (EGF), ciliary growth factor (CNTF), and insulin-like growth factor 1 (IGF1) which promotes a neuroprotective environment in the injury [43].

On the other hand, Th2 helper lymphocytes also promote an increase in the production of growth factors such as BDNF, NGF, neurotrophins 3, 4, and 5 (NT-3; 4 and 5), prevailing an anti-inflammatory microenvironment, supporting cell survival [39] (Figure 3).

## 12. Phagocytic Immune Cells in Acute and Sub-Acute Phases of SCI

The first immune cells to arrive after injury are neutrophils, which will be responsible for the first cleansing of cell debris; these will activate cytokines such as IL-1β, IL-6, TNF-α, inflammation proteins of the macrophages MIP-1α and MIP-1β, proteases, and free radicals, which will favor the activation of glial cells of the CNS [44,45]. Subsequently, there is a recruitment of circulating macrophages and activation of microglia (immune cells of CNS), which begin to phagocyte cell debris. Microglia activates the innate immune response via Toll-like receptors (TLRs). Two polarized states of microglia depending on the signals of the microenvironment there are presented: M1 (pro-inflammatory phenotype) and M2 (anti-inflammatory phenotype), which maintain spinal cord homeostasis [41,46]

The M1 microglia initiates the neurotoxic cascade, activating cytokines such as IL-1β, IL-6, IL-12, TNF-α reactive oxygen species (ROS), iNOS (inducible nitric oxide synthetase), gamma interferon, IFN-γ. These cytokine secretions produce a decrease in cell survival and the promotion of apoptotic cell death [41,44,46,47], (Figure 3).

For the microglia polarization to occur, molecules and signaling pathways are activated that promote changes in the microglia state. The presence of lipocalin 2 (LCN2) at the injury site promotes polarization to M1 (classical activation), which initiates a vicious pro-inflammatory state, other signaling pathways such as activation of NF-κB, p38/MAPK, ERKs, elicits microglia to initiate phagocytic activity, chemotaxis, and expression of pro-inflammatory cytokines, thus clearing the lesion area [45,46,48]. This classic activation causes the activation of proteolytic enzymes that degrade the extracellular matrix, metalloproteinase, collagenase, destroying the integrity of the cells and causing cell death. In addition, they induce neuronal death through the activity of iNOS, thus contributing to secondary degradation and facilitate the recruitment of Th1 lymphocytes [41,45,46,47], (Figure 3).

## 13. The Transition from M1 to M2 Phenotype for Microglia/Macrophage in Sub-Acute and Chronic Phases after SCI

After the increase in the pro-inflammatory environment, there is a change to a protective state in which the microglia phenotype changes to M2, it is divided into three different phenotypes, M2a (Alternative activation), M2b (Alternative activation type II), and M2c (Acquired deactivation) [41,45,46,47].

**Figure 4:**
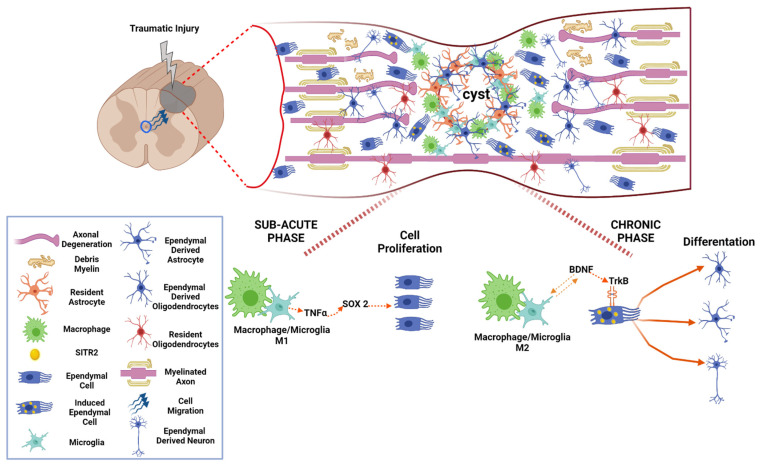
The response of aldynoglia cells in sub-acute and chronic inflammatory phases after SCI. After mechanical damage to the spinal cord, the resident astrocytes near the damaged zone proliferate, enlarge their fibrous processes, produce chondroitin sulfate proteoglycans and form a glial scar containing a cystic cavity in the spinal cord. The ependymal stem cells, EpSCs (a spinal aldynoglia), migrate from the central canal to the injury site; depending on the microenvironment, the EpSCs will proliferate or differentiate. In the sub-acute phase, the TNF-α secreted by macrophages/microglia M1 activates SOX 2, promoting the proliferation of EpSCs. In the chronic phase with the anti-inflammatory microenvironment, the BDNF secreted by macrophages/microglia M2 is attached to the TrkB receptor found in the EpSCs cell surface inducing SITR2 in EpSCs; and promoting its differentiation into astrocytes or oligodendrocytes, which will migrate at fibroglial scar area or remyelinate axons, respectively.

The JAK/STAT pathway acts as a modulator of inflammation, in the presence of anti-inflammatory interleukins such as IL-10 and IL-4, induce the activation and phosphorylation of STAT 3 and STAT 6 via JAK 1, which induces the change to M2 [41,45,46,47]. STAT 3 is recognized based on IL-6 anti-inflammatory and IL-17 by inhibiting NFκB pathways, p38/MAPK producing this change; it has also shown that the peroxisome proliferator-activated receptor gamma (PPARγ) similarly suppresses NFκB signals. Thus, a protective and regenerative environment through the secretion of growth factors such as GDNF, BDNF, cytokines such as IL-10, IL-4, IL-13, TGFβ, as well as the recruitment of Th2 lymphocytes (helper) is also increased. In addition to the proliferation of neural precursor cells, such as aldynoglia cells, of the spinal cord at the site of injury [41,45,46,47], (Figure 3 and Figure 4).

## 14. M1 Macrophage/Microglia Promotes Proliferation of Aldynoglia Cells in Sub-Acute Phase after SCI

Ependymal stem cells (EpSCs) could be considered as a kind of aldynoglia cells in the spinal cord. In the sub-acute phase of SCI, EpSCs migrate to the site of the injury to begin their proliferation and differentiation, in addition to interacting with cells of the immune system (Figure 4). They can differentiate themselves to neurons in vitro, but they differentiate mainly to astroglial phenotype in vivo, suggesting the appearance of aldynoglia cells in this stage. Previous studies have shown that polarized macrophage/microglia (M1/M2) at the injury site directly affects the growth and differentiation of ependymal cells; specifically, M2 polarization promotes differentiation neurons [49,50], (Figure 4).

When the microenvironment at the site of injury is in a pro-inflammatory state, the presence of M1 macrophages/microglia causes a change in ependymal stem cells that migrate to the site of injury. The signals present by the M1 microglia should promote EpSCs proliferation. In this polarization state, the macrophages/microglia triggers self-recovery events by activating Sox2. Sox2 (SRY-box 2) is known to be one of the factors necessary to regulate self-renewal and empowerment in neural stem cell proliferation [49,50], (Figure 4).

It has been shown, in vitro and in vivo, that Sox 2 plays an essential role in the proliferation of EpSCs, due to the presence of cytokines such as TNF-α secreted by the M1 microglia. It activates the signaling pathway of MAPK by activating ERK and P38 kinase, thus promoting Sox 2 regulating cell proliferation in it, and generating new neural cells that can help remedy cell loss [50], (Figure 4).

## 15. M2 Macrophage/Microglia Induce Aldynoglial Cell Differentiation in Chronic Phase of SCI

On the other hand, when this microenvironment changes to an anti-inflammatory M2 phenotype, this makes it clear that proliferating EpSCs can differentiate. The mechanism by which M2 regulates the differentiation of EpSCs is by SIRT2. SIRTs (Sirtuins) Class III NAD^+^ dependent diacetyl histones are related to the catalyzing of various biological processes, such as gene metabolism and expression [49], (Figure 4).

SIRT2 acts directly on regulating the cell cycle, on the response of oxidative stress, and microtubules’ dynamism. In in-vivo and in-vitro work, M2 microglia has been shown to promote up-regulation of SIRT2 in EpSCs, directly affecting their differentiation; this change acts directly on the dynamism of microtubules stabilizing the α-tubulin acetylated EpSCs, thus promoting their differentiation into neurons. This change occurs by growth factors such as BDNF, which is secreted by the M2 microglia, activating the BDNF/TrkB signaling cascade, when the BDNF/TrkB in the EpSCs surface is activated, induces the expression of SIRT2 [49], (Figure 4).

The dimerization and perform the binding of BDNF/TrkB self-phosphorylation of tyrosine-specific residues in the intracellular domains of EpSCs, resulting in phosphotyrosine, which acts as a binding site for protein adaptation, triggers signaling cascades such as MAPKs kinase, phospholipase C range (PLCγ), and phosphatidylinositol 3 (PI3K). When these MAPK kinase pathways are activated by MEK 1/2, it promotes ERK1/2 via Thr-Glu-Tyr, promoting the regulation of SIRT2 deacetylation activity, and finally promoting the differentiation of EpSCs [49,50].

## 16. Chronic Phase of Traumatic Spinal Cord Injury: The Activity of Aldynoglia

In the chronic phase, maturation of fibroglial scar continues, there is demyelination of the axons and own processes of axonal regeneration are also present (Figure 2). When the glial reaction occurs, which results after the injury, there is a recruitment of: microglia, precursors of oligodendrocytes, meninges cells, ependymal cells, and astrocytes site of the injury forming a physical barrier preventing axonal growth, fibroglial scar [33], (Figure 4).

The functions of the glial scar isolate the injured area from the rest of the tissue and produce factors that cause the area to inhibit axonal growth and prevent aberrant connections. The factors present in the glial scar are tenacins, semaphorins, ephrins, and proteoglycans-chondroitin sulfate [51]; these are produced by the cells found in the scar, mainly by reactive astrocytes and oligodendrocytes (Figure 4).

Ependymal stem cells, after injury, migrate to the glial scar and have the ability to differentiate into astrocytes, thus generating a large part of the glial scar. These EpSCs astrocytes do not prevent axonal growth; in contrast, its function is to produce factors that promote neuroprotection, cell replacement, and axonal growth [52]; these described functions belong to aldynoglia cells and reveals their role in the chronic phase after SCI. Also in the chronic phase, a large number of EpSC-derived cells are found in the preserved gray and white matter around the fibrogliotic scar area. These cells are Olig2+ with a morphology of mature oligodendrocytes, which express myelin basic protein while enveloping axons [53] (Figure 4).

## 17. Effect of Aldynoglia Cells on Regeneration after SCI

In traumatic SCI, the initial insult leads to cell damage and begins a cascade of secondary injuries that cyclically lead to neuron and glial cell death, ischemia, and inflammation. These deleterious processes lead to changes in the organization and structural architecture of the spinal cord, including the formation of a glial scar and a cystic cavity. These defensive formations of the injured CNS, in combination with poor endogenous remyelination and weak axonal regrowth, result in poor intrinsic recovery of the spinal cord, such that SCI causes permanent neurologic deficits [54].

However, some regeneration potential is present after SCI; indicating that a permeable and replacement microenvironment occurs at the site of injury. It can activate proteins such as growth-associated protein 43 (GAP-43) or microtubule-associated protein (MAP)1B that initiate axonal growth, but this is insufficient to generate new connections, due to the physical barrier at the injury site [55].

To repair neurologic deficits we can focus on cell transplants as therapy after SCI in order to promote neuroprotection, regeneration, and remyelination and lastly improve morpho-functional recovery [56,57]. Aldynoglia could be a plausible alternative in cell therapy because is a glial cell that promotes axonal growth and remyelination in SCI models [58]. These cells arise from different niches in the brain and spinal cord [5,28,59] (Figure 1) and they have these in common that can differentiate into ensheathing cells, promoting remyelination and axonal out-growth [5,6,8].

The macrophage/microglia response after SCI reflects the type of immune response in subacute and chronic post-injury states. The ratio of M1/M2 macrophages/microglia after injury is related to the balance of the pro/anti-inflammatory response [60]. A change to M2 phagocytic subpopulations seems to favor migration and differentiation in transplanted stem cells and lead to better recovery after SCI [60,61]. A modulatory effect has been observed in the transplanted aldynoglia on the immune cells [62]. In this sense, the potentiation of endogenous (EpSCs) or transplanted aldynoglia in individuals with SCI could improve tissue preservation of white and gray matter and reduce glial scarring.

The potentiation of aldynoglia, identified as EpSCs in models of spinal cord injury, favors axonal regeneration and myelination. This could be obtained by reinforcing the endogenous BDNF/TrkB pathway by transplantation of aldynoglia combined with RhoGTPase inhibitors [3,63]. In addition to maintaining a paracrine effect that could modulate inflammation, it promotes a permissive environment for neuroprotection and axonal growth, as well as proliferation and differentiation within the injury site [59,64]. The process of rewiring by aldaynoglia and neurotrofic factors like BDNF, CNTF, and growth and differentiation factor 10 (GDF10), seems to involve changes in neurons and glial cells of an autonomous system [65,66,67]. It induces axon/dendrite outgrowth and synaptogenesis for reconnection, which occurs in denervated regions such as in the motor and sensory pathways [65]. All these properties are desirable for SCI therapy where functional potentiation of neurons and oligodendrocytes results in better motor recovery.

## 18. Conclusions

Transplantation or endogenous enhancement of spinal aldynoglia becomes an attractive strategy for SCI cell therapy; however, differences in EpSC behavior between human and animal models for SCI must be taken into account. Some efforts to use human CNS-derived stem cells in rodent models for spinal cord injury had failed during preclinical efficacy trials [68]. Delving into the mechanism of action of EpSCs related to their proliferation, migration, and differentiation, as well as the interaction with the host’s immune system, could pave the way for successful therapy after SCI.

## Figures and Tables

**Table 1:** Location in the brain and identity markers of aldynoglia cells.

Location In Brain	Cellular Type	Markers	Bibliography
Subventricular zone, third ventricle	Tanycytes	GFAP, p75 NGFR, vimentin, S100β	[9,10]
Hypothalamus	Pituicytes		
Olfactory Bulb	Ensheathing olfactory cells	GFAP, p75 NGFR, vimentin, S100β, ERα	[11]
Retina	Müller glia cells	GFAP, p75 NGFR, vimentin.	[12,13]
Ventricular area of the cerebellum	Bergmann glia cells	GFAP, p75 NGFR, vimentin, S100β, S0X1, SOX2, SOX9.	[14]
Pineal gland	Pinealocytes	GFAP, vimentin.	[15,16,17]

**Table 2:** Aldynoglia cells in the spinal cord.

Cellular Type	Location	Markers	Bibliography
Ependymocytes	Spinal cord, central canal	nestin, vimentin, S100β	[27,28,29,30]
Tanycytes	Spinal cord, central canal		
